# Household Surgery, or Hints on Emergencies

**Published:** 1850-12

**Authors:** 


*Household Surgery, or Hints on Emergencies.* By JOHN F. SOUTH, one of the Surgeons to St. Thomas's Hospital. *First American, from Second London edition.* Philadelphia: Henry Carey Baird, successor to E. L. Carey, 1850.

This is a reprint of an exceedingly popular English work, which has rapidly exhausted a large edition in its native land. Although very doubtful of the good influence of works on popular medicine and surgery, from the fact that "a little learning is a dangerous thing," we nevertheless believe that the teachings of this book, if followed with the restrictions laid upon the reader by the author, will be found of great assistance to those for whom it was more especially intended, viz., missionaries, captains of vessels, and settlers in colonies where no physician can be had. It is written in a sprightly, and at the same time, intelligible style, and is embellished with many excellent wood cuts, which tell their own story more plainly than words.

